# Hippocampus-based contextual memory alters the morphological characteristics of astrocytes in the dentate gyrus

**DOI:** 10.1186/s13041-016-0253-z

**Published:** 2016-07-26

**Authors:** Moonseok Choi, Sangzin Ahn, Eun-Jeong Yang, Hyunju Kim, Young Hae Chong, Hye-Sun Kim

**Affiliations:** 1 Department of Pharmacology and Biomedical Sciences, College of Medicine, Seoul National University, 103 Daehakro, Jongro-gu 110-799 Seoul, Republic of Korea; 2 Seoul National University College of Medicine, Seoul National University Bundang Hospital, Sungnam, 463-707 Republic of Korea; 3 Department of Microbiology, School of Medicine, Ewha Womans University, 911-1, Mok-6-dong, Yangcheonku Seoul, 158-710 Republic of Korea; 4 Seoul National University College of Medicine, Bundang Hospital, Sungnam, Bundang-Gu, Republic of Korea; 5 Neuroscience Research Institute, College of Medicine, Seoul National University, 103 Daehakro, Jongro-gu Seoul, Republic of Korea

**Keywords:** Astrocyte, Hippocampus-based contextual memory, Morphological changes, GFAP, EAATs, Connexin 43

## Abstract

Astrocytes have been reported to exist in two states, the resting and the reactive states. Morphological changes in the reactive state of astrocytes include an increase in thickness and number of processes, and an increase in the size of the cell body. Molecular changes also occur, such as an increase in the expression of glial fibrillary acidic protein (GFAP). However, the morphological and molecular changes during the process of learning and memory have not been elucidated. In the current study, we subjected Fvb/n mice to contextual fear conditioning, and checked for morphological and molecular changes in astrocytes. 1 h after fear conditioning, type II and type III astrocytes exhibited a unique status with an increased number of processes and decreased GFAP expression which differed from the typical resting or reactive state. In addition, the protein level of excitatory excitatory amino acid transporter 2 (EAAT2) was increased 1 h to 24 h after contextual fear conditioning while EAAT1 did not show any alterations. Connexin 43 (Cx43) protein was found to be increased at 24 h after fear conditioning. These data suggest that hippocampus-based contextual memory process induces changes in the status of astrocytes towards a novel status different from typical resting or reactive states. These morphological and molecular changes may be in line with functional changes.

**Electronic supplementary material:**

The online version of this article (doi:10.1186/s13041-016-0253-z) contains supplementary material, which is available to authorized users.

## Introduction

Astrocytes are well known for the various functions they serve within the central nervous system (CNS) such as metabolic energy support [1–3], maintenance of extracellular ionic homeostasis [4–6], modulation of neurotransmitter actions [7–9] and protection of the CNS from peripheral system via the blood-brain barrier [1, 10, 11]. In addition to these well-known roles of astrocytes, bidirectional communication between astrocytes and neurons has been shown to regulate neuronal excitability and synaptic transmission and plasticity [12–14]. Recent studies are extensively focusing on the roles of astrocytes in synaptic formation and neurogenesis [2, 15, 16], and in supporting learning and memory formation [17, 18]. Morphological changes and synaptic invasion of astrocytes are known to be required for controlling synaptic strength [13]. However, morphological or molecular changes of astrocytes during learning and memory processes still remain unclear.

According to previous reports, astrocytes are classified based on morphology such as cell body size, the number of processes, thickness of processes, direction of processes or length of processes into type I, II and III. Type I astrocytes have a small cell body size and numerous short processes, type II astrocytes have a bipolar shape and long processes. Type III astrocytes are characterized by a star shape and long processes [19–21]. The morphological characteristics of astrocytes are thought to be important for their functions [19, 20].

In this study, we aimed to examine time-dependent morphological and/or molecular changes in astrocytes based on our hypothesis that astrocytes play critical roles in memory formation accompanied by morphological, molecular and functional changes.

Contextual fear conditioning has been exploited to test hippocampal dependent memory in rodents [22–24]. Fvb/n mice were subjected to contextual fear conditioning, and checked for morphological and molecular changes in astrocytes at 1 h and 24 h after contextual fear conditioning. After fear conditioning, type II and type III astrocytes exhibited a unique status with an increased number of processes and decreased protein level of glial fibrillary acidic protein (GFAP) and increased level of excitatory amino acid transporter 2 (EAAT2) and connexin 43 (Cx43) protein which differs from the typical resting or reactive state.

These results show that hippocampus-based contextual memory processes results in changes in the status of astrocytes towards a novel status different from typical resting or reactive states. These morphological and molecular changes may be in line with functional changes.

## Results

### Fvb/n mice were exposed to contextual fear conditioning

**Fig. 1:**
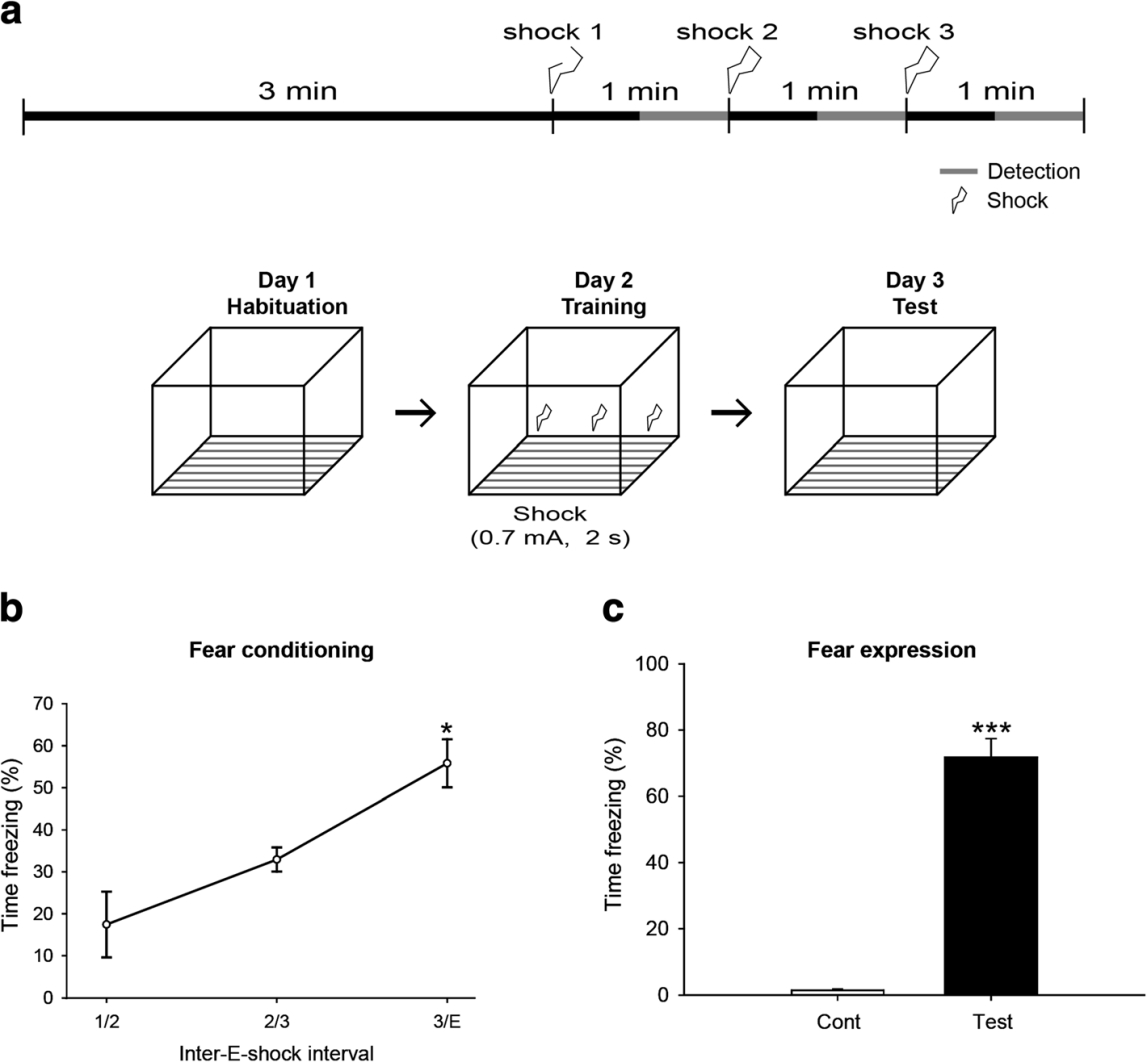
Fvb/n mice were exposed to contextual fear conditioning. **a** Illustration of the scheme for the contextual fear conditioning test (**b**) At day 2, mice displayed increased freezing behavior at 1^st^ and 2^nd^ electric shocks (1/2), the 2^nd^ and 3^rd^ (2/3) and after the 3^rd^ electric shocks (3/E) 1/2, 2/3 and 3/E, (17.43 ± 7.86 %, 32,95 ± 2.88 % and 55.83 ± 5.72 %), respectively, indicating that the mice were well trained by the fear conditioning process. **c** At day 3, the test group exhibited significantly increased time freezing (71.786 ± 3.84 %, ***p < 0.001), compared to the control group (4.817 ± 0.447 %), indicating that contextual fear memory was induced by our contextual fear conditioning protocol. *p < 0.05 compared to shock 1 (**b**) or control (**c**). Data are presented as the mean ± SEM. Cont, control group

We used contextual fear conditioning for expressing hippocampal dependent long-term spatial memory. Generally, a conditioned stimulus (CS) and an unconditioned stimulus (US) are used for this behavior test. However, we employed contextual conditioning without a sound cue (CS) because we aimed to focus only on the hippocampus-dependent circuit. A schematic time-schedule of the fear conditioning we used in this study is shown in Fig. 1a.

We confirmed that at intervals between the 1^st^ and 2^nd^ electric shocks (1/2), the 2^nd^ and 3^rd^ (2/3) and after the 3^rd^ electric shocks (3/E), mice displayed increased freezing behavior at rates of 17.43 ± 7.86 %, 32,95 ± 2.88 % and 55.83 ± 5.72 %, respectively, indicating that the mice were well-trained by the fear conditioning (Fig. 1b). At day 3, the freezing behavior of the control and the test group were tested. The test group exhibited significantly increased freezing time (71.786 ± 3.84 %, *p* < 0.001, by a Student’s *t*-test), compared to the control group (4.817 % ± 0.447, Fig. 1c). These data indicated that contextual fear memory was induced by the contextual fear conditioning protocol.

### Alterations in the astrocytic morphological characteristics were observed in the hippocampal dentate gyrus after contextual fear conditioning

Confocal images of hippocampal slices from the 2 group mice were taken with Z-stack at 0 ~ 30 μm and reconstructed into 3-D images. The number of intersections between astrocyte processes and concentric circles were manually counted.

**Fig. 2:**
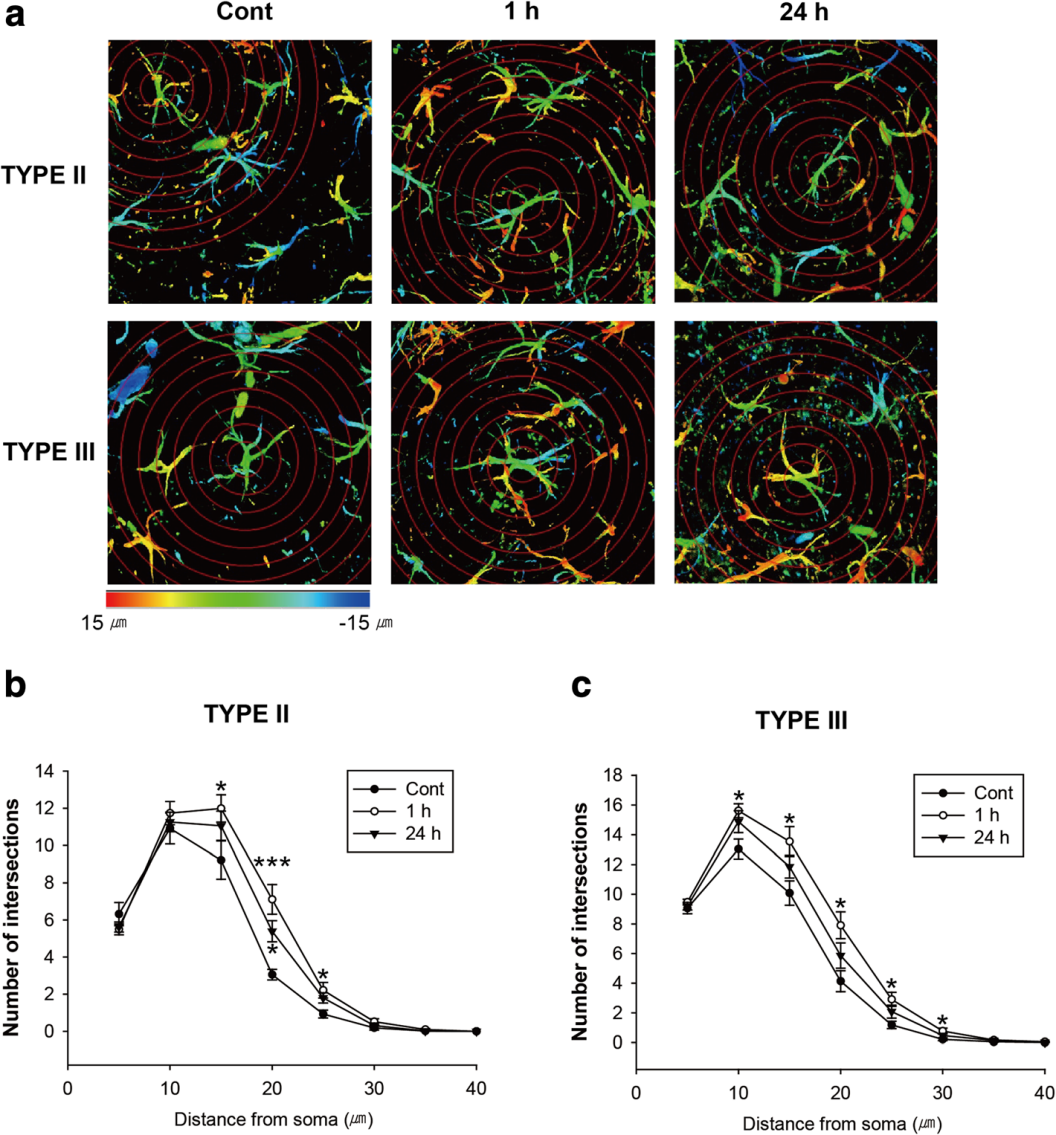
Morphological changes in astrocytes were detected in the dentate gyrus. **a** Morphological analysis of type II and type III astrocytes by sholl analysis. (Immunofluorescence for GFAP, gradation from red to blue; depth of Z-projection is 0-30 μm, Concentric circles are spaced at 10 μm). **b** Number of intersections between each circle and processes of type II astrocytes in dentate gyrus from control, 1 h and 24 h groups (Control, *N* = 9, *n* = 34; 1 h, *N* = 10, *n* = 40; 24 h, *N* = 8, *n* = 56). **c** Number of intersections between each circle and processes of type III astrocyte in the dentate gyrus from control, 1 h and 24 h group (Control, *N* = 10, *n* = 111; 1 h, *N* = 12, *n* = 155; 24 h, *N* = 10, *n* = 117). *p < 0.05, ***p < 0.001. GFAP, glial fibrillary acidic protein

In humans, patients with amnestic mild cognitive impairment show shape and volume changes in the CA3 and dentate gyrus of the hippocampus [25]. Therefore, the dentate gyrus is one of the important regions for long-term memory and was chosen as the focus of this study. Type I astrocytes were found to constitute only a small percentage of astrocytes in the dentate gyrus (data not shown). The intersections of type II astrocytes and thus the processes of these cells, were significantly increased at 15 μm from the cell body at 1 h but not at 24 h after fear conditioning was performed (control: 9.216 ± 1.023, 1 h: 11.988 ± 0.737 (*p* < 0.05). At 20 μm, the number of intersections were significantly increased at both 1 h and 24 h (control; 3.056 ± 0.285, 1 h; 7.106 ± 0,798 (*p* < 0.001), 24 h 5.391 ± 0.569 (*p* < 0.05). At 25 μm, the number of intersections was significantly increased at 1 h (control: 0.929 ± 0.0213, 1 h; 2.217 ± 0.420, *p* < 0.01)) (Fig. 2b).

The intersections of Type III astrocytes were significantly increased at 10 μm from the cell body at 1 h but not at 24 h (control; 13.036 ± 0.669, 1 h; 15.618 ± 0.473, *p <* 0.01). At 15 μm, the number of intersections was significantly increased at 1 h but not at 24 h (control; 10.083 ± 0.821, 1 h 13.543 ± 0.998, *p* < 0.05). At 20 μm, the number of intersections was also significantly increased at 1 h but not at 24 h (control, 4.132 ± 0.704, 1 h; 7.906 ± 0.908, *p* <0.05). At 25 μm, the number of intersections was significantly increased at 1 h but not at 24 h (control; 1.184 ± 0.248, 1 h; 2.905 ± 0.476, *p* <0.05). At 30 μm, the number of intersections was significantly increased at 1 h but not at 24 h (control; 0.207 ± 0.081, 1 h 0.767 ± 0.199, *p* < 0.05) (Fig. 2c). These data indicate that hippocampal dependent memory induction causes the changes in the number of processes of Type II and Type III astrocytes in the dentate gyrus at 1 h after a test of fear conditioning. Meanwhile, the intersections of Type III astrocytes were not changed in the auditory cortex (Additional file 1: Figure S1). In this area, the numbers of Type I and II astrocytes were much smaller than Type III astrocytes, unlike in the dentate gyrus (data not shown)

### Protein levels of GFAP, PSD-95, EAAT2 and Cx43 were altered in the hippocampus by contextual fear conditioning

**Fig. 3:**
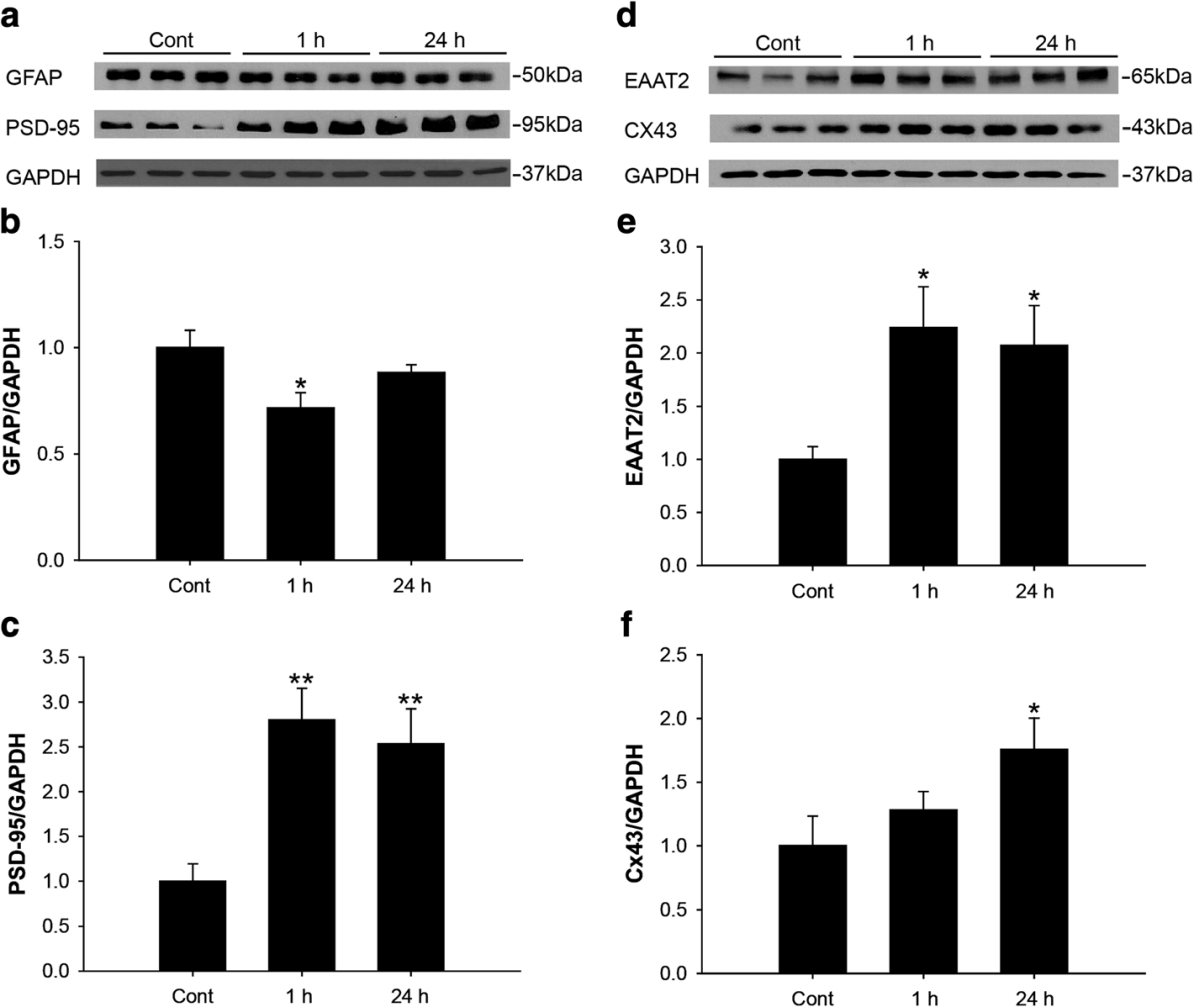
Protein levels of GFAP, PSD-95, EAAT2 and CX43 were altered in the hippocampus by contextual fear conditioning. **a** Representative immunoblots for GFAP, PSD-95 and GAPDH for control, 1 h and 24 h groups (*n* = 9). **b** Densitometric analysis of immunoblots for GFAP (*n* = 9) (**c**) Densitometric analysis of immunoblots for PSD-95 (*n* = 6) (**d**) Immunoblots for EAAT2, Cx43 and GAPDH for control, 1 h and 24 h groups (*n* = 6). **e** Densitometric analysis of immunoblots for EAAT2 (*n* = 6) (**f**) Densitometric analysis of immunoblots for Cx43 (*n* = 6). *p < 0.05, **p < 0.01

Next, we analyzed the protein levels of GFAP, PSD-95, EAAT1, EAAT2 and Cx43 to investigate the molecular changes accompanied by morphological changes in astrocytes induced by hippocampal contextual memory formation. We found that the protein levels of GFAP, a reactive astrocyte marker, were significantly decreased in the hippocampus of the test group at 1 h after the conditioning test compared with the control group (control, 1.000 ± 0.8198, 1 h, 0.7173 ± 0.07201, *p* < 0.05, Figs. 3a and b).

Long-term memory induction strengthened synaptic connectivity of hippocampal neurons. We investigated the protein levels of PSD-95, a major post-synaptic scaffolding protein, in the hippocampus after fear conditioning was performed [26]. PSD-95 protein level was significantly increased at 1 h and at 24 h (control; 1.000 ± 0.193, 1 h; 2.800 ± 0.352, *p* <0.01, 24 h; 2.535 ± 0.388, *p* <0.05 Figs. 3a and c).

The protein level of EAAT2, glutamate transporter, and specifically expressed in astrocytes [27, 28], was increased at 1 h and 24 h (control; 1.000 ± 0.121, 1 h; 2.238 ± 0.386, *p* <0.01, 24 h; 2.070 ± 0.377, *p* <0.05 Figs. 3d and e), while EAAT1 did not show any changes (data not shown). Cx43 is a major gap junction protein that connects astrocytes. Recently, it has been reported that Cx43 not only serves as a gap junction protein but also functions as a hemichannel to affect neuron functions during inflammation [29]. The protein level of Cx43 was not significantly increased at 1 h in the test group. But it was increased at 24 h (control; 1.000 ± 0.233, 1 h; 1.280 ± 0.146, *p* =0.116, 24 h; 1.759 ± 0.245, *p* <0.05 Figs. 3d and f). Taken together, these data suggest that during the formation of contextual fear memory formation, astrocytes change dynamically with reduced protein levels of GFAP and increased levels of EAAT2 and Cx 43 proteins. This appears to be a different status of astrocytes from the typical resting or reactive states.

### LAA injection into the dentate gyrus inhibited the contextual fear memory expression after the fear conditioning

**Fig. 4:**
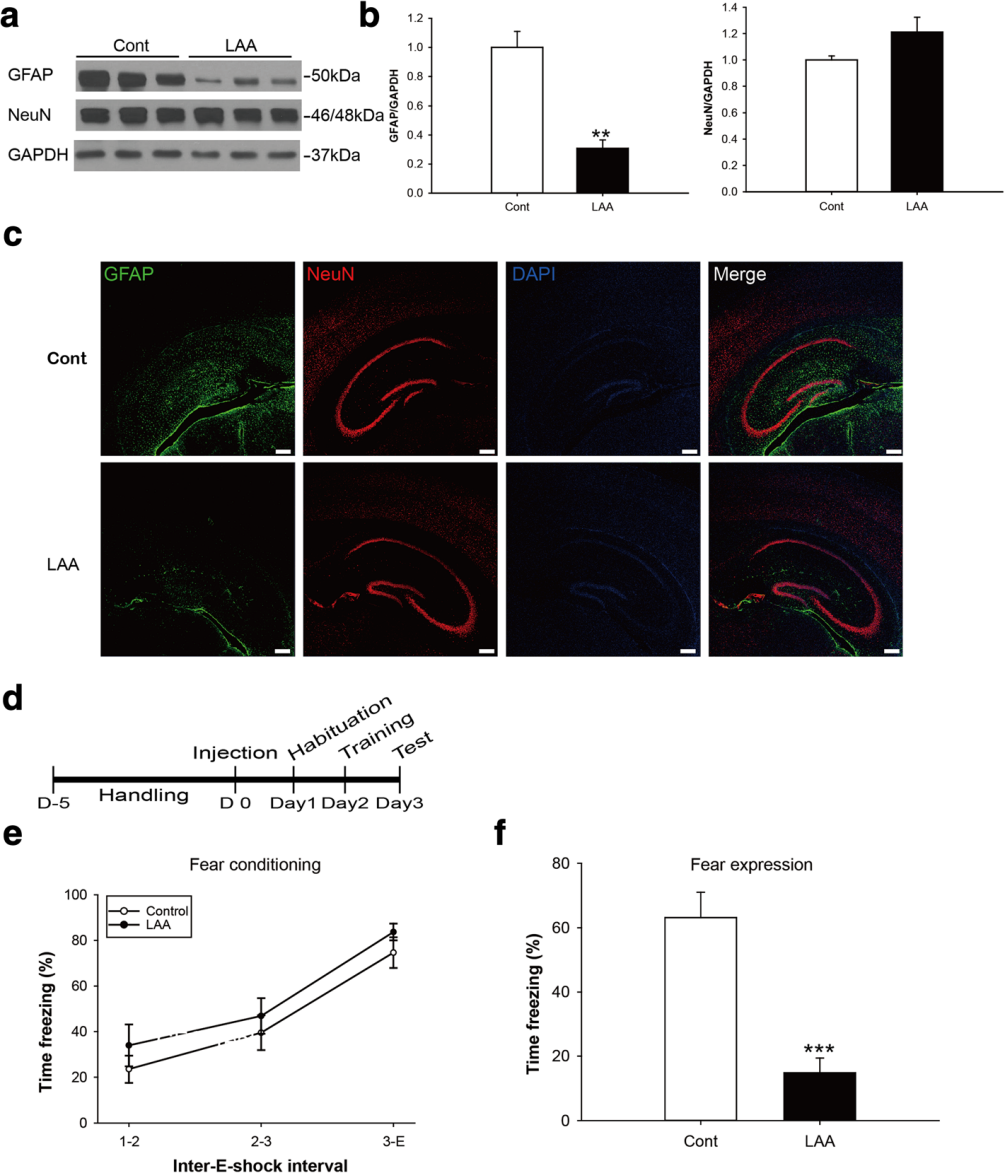
LAA injection in the dentate gyrus reduced the expression of fear memory after contextual fear conditioning (**a**) Representative immunoblots for GFAP, NeuN and GAPDH in hippocampus for the control and LAA groups (*n* = 5). **b** Densitometric analysis of immunoblots for GFAP and NeuN (*n* = 5). **c** Immunofluorescence for GFAP, NeuN and DAPI in hippocampus for the control and LAA groups. **d** Schedule for contextual fear conditioning with control and LAA- injected mice. **e** During fear conditioning, the control and LAA groups displayed increased time freezing at 1^st^ and 2^nd^ electric shocks (1/2), the 2^nd^ and 3^rd^ (2/3) and after the 3^rd^ electric shocks (3/E) (*n* = 10). **f** During fear expression, after 24 h the electric shock, the control and LAA groups showed increased time freezing (*n* = 10)

L-α-aminoadipate (LAA), a glutamate analogue, has been generally accepted to exert astrocyte-specific toxicity, although the exact mechanism is not fully elucidated [30–32]. We injected 20 μg of LAA into the dentate gyrus via stereotaxic surgery. Forty-eight hours after the injection, we evaluated the protein levels of GFAP and NeuN via Western blotting and immunohistochemistry (Figs. 4a, b and c). We confirmed that the protein level of GFAP was significantly decreased by LAA injection by approximately 70 % (control; 1.000 ± 0.11, LAA; 0.307 ± 0.058, *p* <0.01 Figs. 4a and b) whereas that of NeuN was not significantly altered. This result was also confirmed with immunohistochemistry (Fig. 4c). These data indicate that LAA injection into the dentate gyrus specifically reduced the numbers of astrocytes but not of neurons (Fig. 4c).

We performed contextual fear conditioning with control and LAA-injected groups. The timetable for LAA injection and contextual conditioning is shown in Fig. 4d. There was no difference in the time freezing between the control and LAA-injected group during fear conditioning (Fig. 4e). However, in the test, 24 h after fear conditioning, the LAA group showed significantly decreased time freezing compared to the vehicle-treated control group (control; 63.169 % ± 7.911 %, LAA; 14.784 % ± 4.610 %, *p* <0.001 Figs. 4f). We analyzed the motor functions in both the control and the LAA-injected group during the habituation process. Both groups did not show any significant difference in the total distance travelled and the velocity (Additional file 2: Figure S2).

## Discussion

In the present study, we investigated the morphological and molecular changes in astrocytes based on our hypothesis that these changes are accompanied by long-term memory induction in the dentate gyrus of the hippocampus. We exposed Fvb/n mice to contextual fear conditioning, and checked for morphological and molecular changes in astrocytes. We found that 1 h after fear conditioning, type II and type III astrocytes exhibit a unique status with an increased number of processes and decreased protein level of GFAP which differ from the typical resting or reactive states. The reactive state of astrocyte is known to show increased GFAP expression level [33]. However, our results showed that hippocampus-based contextual memory induced increased intersections of type II and type III astrocytes but decreased GFAP protein levels were observed. In addition, we also found that the protein levels of EAAT2 and Cx43 were increased. Previously, it has been reported that physical exercise improved learning and memory capability and induced an increase in the number of astrocyte processes [34]. Taken together, these results suggest that an altered astrocytic state is necessary to induce the learning and memory process. In a recent MRI study in humans and rodents, Sagi et al. showed that the complexity of the hippocampal dentate gyrus was increased after acquisition of spatial memory [35]. In addition, the intensity of GFAP protein was increased in the hippocampus in an immunofluorescence study.

In our study, we used confocal laser scanning microscopy. A live imaging system such as two-photon confocal laser scanning microscopy is needed to observe dynamic morphological changes of hippocampal astrocytes during contextual memory formation.

The increased number of astrocyte processes indicates an increase in the number of tripartite synapses. In addition, other studies have demonstrated that improved learning and memory capability are accompanied by the increased number of astrocyte processes. Further study with high resolution, such as at the electron microscope level is needed to image invasion into tripartite synapse structure. Additionally the mechanism between the increased number of astrocyte processes and decreased expression of GFAP observed in the current study remains unclear.

Enhanced synaptic plasticity, including increased amplitude and fEPSP slope during LTP induction was previously reported in the CA1 region of GFAP-null mice [36]. It is thought that GFAP is related to the learning and memory process.

LAA is well known to exert astrocyte-specific toxicity. Based on previous report, 20 μg of LAA was injected into the dentate gyrus to reduce the number of astrocytes in this area [37]. We confirmed that LAA group showed decreased time freezing. These data suggest that the presence of astrocyte is critically required for the formation of long-term memory.

Collectively, the increased numbers of astrocyte processes and the decreased GFAP expression level in the dentate gyrus are correlated for hippocampal dependent long-term memory induction. These morphological and biological changes, i.e., the altered protein level of GFAP was returned to the control level at 24 h. The characteristics of astrocytes observed in the current study are different from the resting or reactive states of astrocytes. Thus, a dynamic change from the memory induction state astrocyte is correlated with the induction of long-term memory.

These results show that hippocampus-based contextual memory process results in the changes in the status of astrocytes towards a novel status different from typical resting or reactive states. These morphological and molecular changes may be in line with functional changes.

## Methods

### Reagents and antibodies

RNA and protein isolation kit were purchased from NucleoSpin (#740933.50, Macherey-Nagel, Dűren, Germany) and Pierce™ BCA Protein Assay Kit were purchased from Thermo (#23227, MA, USA). Anti-GFAP, rabbit polyclonal antibody was purchased from DAKO (#Z0334, CA, USA). Anti-Iba1, rabbit polyclonal antibody was purchased from WAKO (#016-20001, Osaka, Japan). Anti-mouse, sheep polyclonal horseradish peroxidase (HRP) tagged antibody was purchased from Abcam (#ab26116, #ab26113 and #ab6808, EA, UK). Anti-EAAT2 rabbit monoclonal antibody was purchased from Cell signaling (#3838, MA, USA). Anti-Cx43 mouse monoclonal and anti-EAAT1 antibodies were purchased from Santacruz (#sc-59949 and #sc-15316, Taxas, USA). Anti-NeuN rabbit monoclonal antibody was purchased from Millipore (#3838, MA, USA). Anti-GAPDH, rabbit polyclonal antibody was purchased from AbFrontier (#LF-PA0018, Seoul, Korea). Anti-PSD-95, mouse monoclonal antibody and anti-rabbit, goat polyclonal tagged alexa fluor 488 and 4’.6-diamidino-2-phenylinodole (DAPI) were purchased from ThermoFisher (#MA1-046, #A11034, #A11012 and #D3571, MA, USA).

### Experimental animals

FVB/N mice were obtained from Central Laboratory Animal Incorporation (Seoul, Korea) and the 6 to 8 week-old male mice were used for the experiments. The mice were housed in group of five per cage with a 12 h light/dark cycle and ad libitum access to food and water as under standard laboratory housing condition.

### Contextual fear conditioning

Contextual fear conditioning was tested as described previously [38, 39]. Each scrambler was connected to an electronic constant-current shock source that was controlled via an interface connected to a Windows 7 computer running EthoVision XT 8 software (Noldus Information Technology, VA, USA). A digital camera was mounted on the steel ceiling of each chamber, and video signals were sent to the same computer for analysis. Prior to training, mice were placed into the chamber for 60 min for habituation. During training, mice were placed in the conditioning chamber (13 × 13 × 25 cm) for 3 min (for pre-shock) and received three repetitions of a foot-shock (0.7 mA, 2 sec) at 1 min inter-trial intervals. On the next day, conditioned mice were placed in the same chamber, and the “freezing” time was measured over periods of 3 min. Conditioned freezing was defined as immobility except for respiratory movements. The total freezing time in the test period was represented as a percentage. Animals were divided into 2 groups. The sham control group (control group) did not receive electric foot shocks even though all other the fear conditioning procedures were the same as the test group. The test group was exposed to contextual fear conditioning with electric foot shock on day 2 (Fig. 1a).

### Western blotting

For brain tissue preparation, mice were deeply anesthetized with Zoletil (12.5 mg/kg) and Rompun mix (17.5 mg/kg) administered intraperitoneally. Mice were perfused transcardially with heparin dissolved in PBS (pH 7.2). The dissected brain tissues were frozen at -80 °C for Western blotting. Tissues were homogenized with total RNA and protein isolation kit (NucleoSpin® RNA/Protein #740933.50, Macherey-Nagel, Dűren, Germany). The protein samples were quantified with Pierce™ BCA Protein Assay Kit and 50 μg protein sample were used for each Western blot. The primary antibodies were applied in the following concentrations: anti-GFAP (rabbit, 1: 1,000; Dako #Z0334), anti-Iba1 (rabbit, 1:300; Wako #NB100-1028), anti-EAAT1 (rabbit, 1:1,000; Santacruz # sc-15316), anti-EAAT2 (rabbit, 1:1,000; Cellsignaling #3838), anti-Cx43 (mouse, 1:1,000; Santacruz #sc-59949), anti-PSD95 (mouse, 1:2,000; Thermo #MA1-046), anti-NeuN (mouse, 1:1,000; Millipore #MAB377) anti-GAPDH (rabbit, 1:10,000; Abfrontier #LF-PA0018). Secondary antibodies were conjugated with horse-radish peroxidase (HRP) (1: 10,000, Invitrogen). The HRP signals were visualized using an enhanced chemiluminescent (ECL, Abfrontier #LF-QC0101, Gyeonggi-do, Korea) substrate.

### Immunohistochemistry

Brains were removed and perfused transcardially with heparin dissolved in PBS (pH 7.2) for 5 min. Brains were then perfused with 4 % paraformaldehyde in PBS for 5 min, and fixed in a 4 % paraformaldehyde for 24 h at 4 °C and incubated in 30 % sucrose solution for 48 h at 4 °C. For immunohistochemical experiments, brain tissues were coronal sectioned 30 μm with a Crystat (Cryotome, Thermo Elctron Corporation, MA, USA) and stored in cryoprotection solution at 4 °C. For antigen retrieval, brain sections were incubated with 10 mM sodium citrate (pH 8.5) in an 80 °C water bath for 30 min. Brain sections were then blocked with 0.3 % triton X-100, 2 % horse serum and 2 % BSA in PBS for 1 h. The anti-GFAP (rabbit) antibody was applied at a concentration of 1: 1,000 (Dako #Z0334). Secondary antibodies were applied in the following concentrations; anti-rabbit-488 (goat, 1:200; Invitrogen #A11034) and 1 μM DAPI. Sections were mounted in microscope slides in a mounting solution (DAKO). Confocal microscopic observation was performed using LSM 510 (Carl Zeiss, Germany).

### Analysis of astrocytes morphology

Sholl analysis was performed to investigate the changes in astrocytes morphology as previously described [40]. Astrocytes were stained with anti-GFAP antibody in the hippocampal dentate gyrus and the images were obtained every 1 μm interval between 0 to 30 μm depth with Z-Stack system using LSM 510 confocal microscope. Confocal Z-stacks were reconstructed in 3-D using LSM software and sholl analysis was performed with the 3-D reconstructed confocal image. Concentric circles with 10 um diameters were drawn from the center of the cell body of an astrocyte. Processes intersecting the concentric circles were manually counted at each distance from the cell body by adobe Photoshop CC 2014. This manual counting method allowed analysis of the finest astrocyte processes (1 pixel or more in size).

### Stereotaxic surgery for LAA injection

Stereotaxic surgery was performed as described previously [41]. All the mice were deeply anesthetized with isoflurane. LAA (20 μg) was bilaterally injected via a Hamilton syringe into the dentate gyrus of the hippocampus. (AP - 1.5, ML ± 1.4, DV - 3.1 at a rate of 0.15 um/min up to 1 μl).

### Statistical analysis

Data are expressed as the mean ± SEM (means ± standard error of the mean). One-way ANOVA followed by Fisher’s LSD post-hoc analysis or Students’ *t*-test (SPSS, IL, USA) was performed to determine statistical significance. The results were considered to be statistically significant at *p* < 0.05.

## Abbreviations

CNS, central nerve system; CS, a conditioned stimulus; Cx43, connexin 43; EAAT, excitatory amino acid transporter; GFAP, glial fibrillary acidic protein; LAA, L-α-aminoadipate; NeuN, neuronal nuclei; US, an unconditioned stimulus

## Additional files

Additional file 1: Figure S1. Morphological changes in astrocytes were examined in the auditory cortex. (A) Morphological analysis of type III astrocytes by sholl analysis. (Immunofluorescence for GFAP, gradation from red to blue; depth of Z-projection is 0-30 μm, Concentric circles are spaced at 10 μm). (B) Number of intersections between each circle and processes of type III astrocytes in auditory cortex from control, 1 h and 24 h groups (Control, *N* = 5, *n* = 15; 1 h, 6 = 10, *n* = 22; 24 h, 5 = 8, *n* = 16). (TIF 3128 kb) Additional file 2: Figure S2. LAA injection did not affect the motor function. (A) The total distance was examined for both control and LAA group with auto-tracking system in Ethovision. (*n* = 10). (B) The velocity was calculated for the control and LAA groups with the auto-tracking system in Ethovision (*n* = 10). (TIF 205 kb)
