# Supplementary figures and images for: Hippocampus-based contextual memory alters the morphological characteristics of astrocytes in the dentate gyrus
Source: Mol Brain. 2016 Jul 26;9:72. doi: 10.1186/s13041-016-0253-z (PMC4962445; doi:10.1186/s13041-016-0253-z)

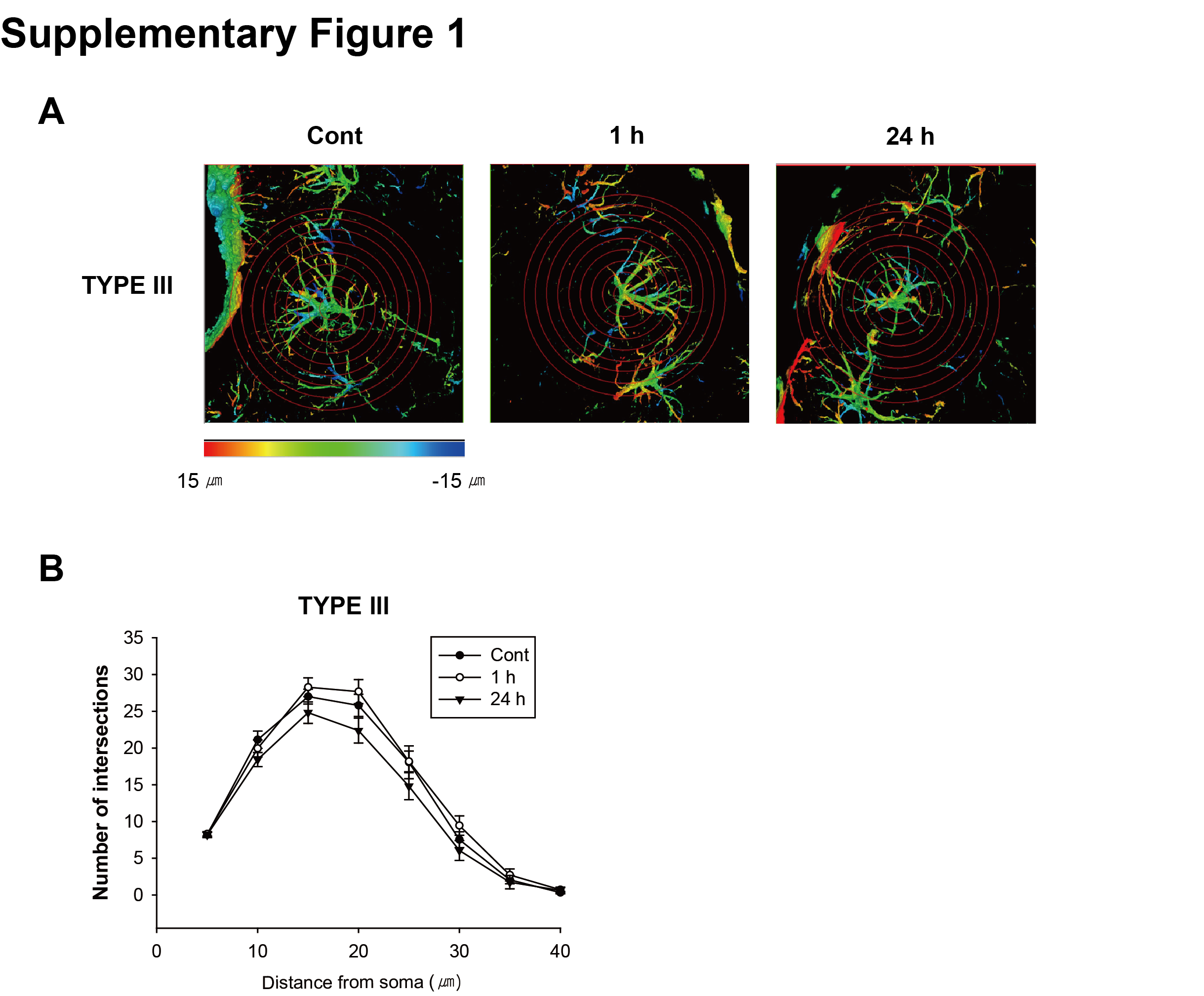

Supplement: Additional file 1: Figure S1. — Morphological changes in astrocytes were examined in the auditory cortex. (A) Morphological analysis of type III astrocytes by sholl analysis. (Immunofluorescence for GFAP, gradation from red to blue; depth of Z-projection is 0-30 μm, Concentric circles are spaced at 10 μm). (B) Number of intersections between each circle and processes of type III astrocytes in auditory cortex from control, 1 h and 24 h groups (Control, N = 5, n = 15; 1 h, 6 = 10, n = 22; 24 h, 5 = 8, n = 16). (TIF 3128 kb) [file 13041_2016_253_MOESM1_ESM.tif]

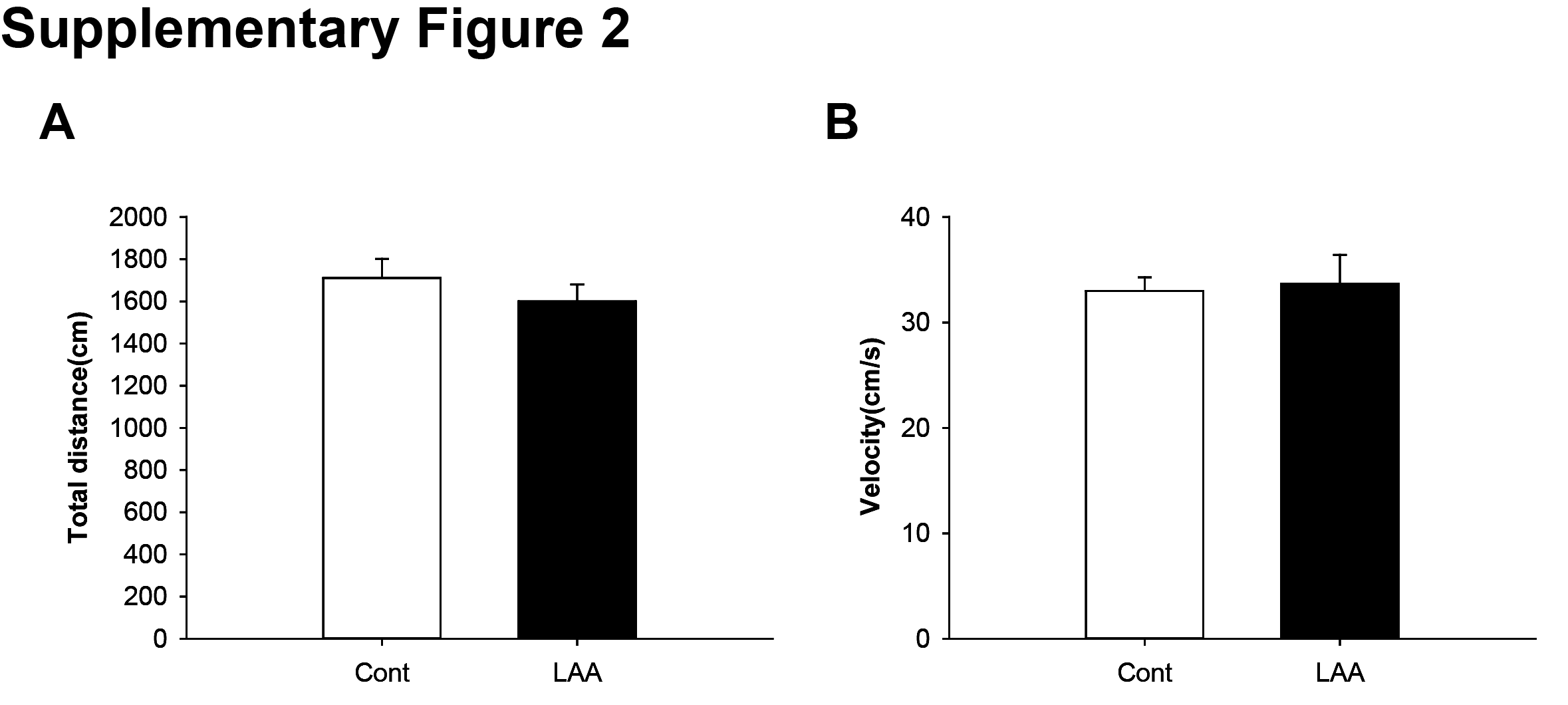

Supplement: Additional file 2: Figure S2. — LAA injection did not affect the motor function. (A) The total distance was examined for both control and LAA group with auto-tracking system in Ethovision. (n = 10). (B) The velocity was calculated for the control and LAA groups with the auto-tracking system in Ethovision (n = 10). (TIF 205 kb) [file 13041_2016_253_MOESM2_ESM.tif]
